# Supplementary material for: Comparison of Rapid Nucleic Acid Extraction Methods for SARS-CoV-2 Detection by RT-qPCR
Source: Diagnostics (Basel). 2022 Feb 27;12(3):601. doi: 10.3390/diagnostics12030601 (PMC8946922; doi:10.3390/diagnostics12030601)
Supplement: Supplementary file 1 [file diagnostics-12-00601-s001.zip › diagnostics-1600755-supplementary.pdf]

diagnostics

MDPI

SUPPLEMENTARY MATERIAL

Article

Comparison of rapid nucleic acid extraction methods for SARS-CoV-2 detection by RT-qPCR

Lívia Mara Silva[1], Lorena Rodrigues Riani[1], Marcelo Silva Silvério[1], Olavo dos Santos Pereira-Junior[1], Frederico Pittella[1*]

[1] Faculdade de Farmácia, Universidade Federal de Juiz de Fora, Brazil.
* Correspondence: Frederico Pittella. E-mail: frederico.pittella@ufjf.edu.br.

Supplementary Table S1 - RT-qPCR results of reactions performed with Allplex kit after extraction with PureLink RNA Mini Kit (original Ct values used for diagnosis).

ID	Medium	Extraction	CT Value Gene N	CT Value Gene E	CT Value Gene RdRp	Result
S1	VTM	Column	22.2	19.3	20.8	Detected
S2	VTM	Column	27.9	24.6	26.7	Detected
S3	VTM	Column	26.0	24.0	26.0	Detected
S4	VTM	Column	Undetermined	Undetermined	Undetermined	Not detected
S5	VTM	Column	28.3	24.9	26.7	Detected
S6	VTM	Column	26.1	23.3	25.4	Detected
S7	VTM	Column	32.6	29.5	31.4	Detected
S8	VTM	Column	Undetermined	Undetermined	Undetermined	Not detected
S9	VTM	Column	Undetermined	Undetermined	Undetermined	Not detected
S10	VTM	Column	23.2	19.8	21.6	Detected
S11	VTM	Column	26.5	23.5	25.9	Detected
S12	VTM	Column	Undetermined	Undetermined	Undetermined	Not detected
S13	VTM	Column	26.4	24.2	26.7	Detected
S14	VTM	Column	Undetermined	Undetermined	Undetermined	Not detected
S15	VTM	Column	31.0	28.2	31.0	Detected
S16	VTM	Column	22.0	19.8	21.5	Detected
S17	VTM	Column	Undetermined	Undetermined	Undetermined	Not detected
S18	VTM	Column	30.5	28.3	30.6	Detected
S19	VTM	Column	Undetermined	Undetermined	Undetermined	Not detected
S20	VTM	Column	Undetermined	Undetermined	Undetermined	Not detected
S21	VTM	Column	Undetermined	Undetermined	Undetermined	Not detected
S22	VTM	Column	16.6	15.9	17.8	Detected
S23	VTM	Column	Undetermined	Undetermined	Undetermined	Not detected
S24	VTM	Column	27.5	24.8	26.8	Detected
S25	VTM	Column	30.4	27.2	29.1	Detected
S26	VTM	Column	32.6	19.4	31.4	Detected
S27	VTM	Column	24.1	20.7	22.7	Detected

S28	VTM	Column	24.3	20.8	22.3	Detected
S29	VTM	Column	24.0	21.8	23.5	Detected
S30	VTM	Column	22.4	21.1	22.2	Detected
S31	VTM	Column	23.2	19.5	21.3	Detected
S32	VTM	Column	19.1	16.1	17.1	Detected
S33	VTM	Column	Undetermined	Undetermined	Undetermined	Not detected
S34	VTM	Column	Undetermined	Undetermined	Undetermined	Not detected
S35	VTM	Column	21.4	19.0	20.0	Detected
S36	VTM	Column	30.6	28.2	30.0	Detected
S37	VTM	Column	39.0	38.6	Undetermined	Detected
S38	VTM	Column	29.8	26.7	28.8	Detected
S39	VTM	Column	Undetermined	Undetermined	Undetermined	Not detected
S40	VTM	Column	Undetermined	Undetermined	Undetermined	Not detected
S41	VTM	Column	Undetermined	Undetermined	Undetermined	Not detected
S42	VTM	Column	28.5	25.7	27.8	Detected
S43	VTM	Column	Undetermined	Undetermined	Undetermined	Not detected
S44	VTM	Column	22.4	21.1	22.2	Detected
S45	Saline	Column	Undetermined	Undetermined	Undetermined	Not detected
S46	Saline	Column	Undetermined	Undetermined	Undetermined	Not detected
S47	Saline	Column	19.8	17.0	18.7	Detected
S48	Endotracheal aspirate	Column	22.6	19.8	21.6	Detected
S49	Endotracheal aspirate	Column	Undetermined	Undetermined	Undetermined	Not detected
S50	Endotracheal aspirate	Column	Undetermined	Undetermined	Undetermined	Not detected

CT rt-qPCR = Cycle threshold

Supplementary Table S2 - RT-qPCR results of reactions performed with Bio-rad kit and IDT primers-probes after extraction with PureLink RNA Mini Kit (original Ct values used for diagnosis).

ID	Medium	Extraction	CT Value Gene N1	CT Value Gene N2	CT Value Gene RNAseP	Result
S51	VTM	Column	23.5	22.9	26.9	Detected
S52	VTM	Column	30.6	29.4	28.8	Detected
S53	VTM	Column	Undetermined	Undetermined	28.6	Not Detected
S54	VTM	Column	Undetermined	Undetermined	28.8	Not Detected
S55	Saline	Column	Undetermined	Undetermined	28.4	Not Detected
S56	Saline	Column	Undetermined	Undetermined	28.7	Not Detected
S57	Saline	Column	19.7	18.6	27.7	Detected
S58	Endotracheal aspirate	Column	21.8	21.3	27.3	Detected
S59	Endotracheal aspirate	Column	Undetermined	Undetermined	26.8	Not Detected
S60	Endotracheal aspirate	Column	Undetermined	Undetermined	26.4	Not Detected

CT rt-qPCR = Cycle threshold
